# Is ecological speciation a major trend in aphids? Insights from a molecular phylogeny of the conifer-feeding genus *Cinara*

**DOI:** 10.1186/1742-9994-10-56

**Published:** 2013-09-18

**Authors:** Emmanuelle Jousselin, Astrid Cruaud, Gwenaelle Genson, François Chevenet, Robert G Foottit, Armelle Cœur d’acier

**Affiliations:** 1 INRA–UMR 1062 CBGP (INRA, IRD, CIRAD, Montpellier SupAgro), Centre de Biologie pour la Gestion des Populations, Campus International de Baillarguet CS 30 016, F-34 988, Montferrier-sur-Lez, France; 2 Institut de Biologie Computationnelle, LIRMM, UMR 5506 CNRS–Université Montpellier 2, Montpellier, France; 3 MIVEGEC, CNRS 5290, IRD 224, Universités Montpellier 1 et 2, Montpellier, France; 4 Agriculture and Agri-Food Canada, Canadian National Collection of Insects, K.W. Neatby Building, 960 Carling Avenue, Ottawa, ON K1A 0C6, Canada

**Keywords:** Ecological speciation, Niche shifts, Host shift, Host race, Geographic isolation, Phytophagous insect, Species delimitation, Cladogenesis, DNA phylogeny

## Abstract

**Introduction:**

In the past decade ecological speciation has been recognized as having an important role in the diversification of plant-feeding insects. Aphids are host-specialised phytophagous insects that mate on their host plants and, as such, they are prone to experience reproductive isolation linked with host plant association that could ultimately lead to species formation. The generality of such a scenario remains to be tested through macroevolutionary studies. To explore the prevalence of host-driven speciation in the diversification of the aphid genus *Cinara* and to investigate alternative modes of speciation, we reconstructed a phylogeny of this genus based on mitochondrial, nuclear and *Buchnera aphidicola* DNA sequence fragments and applied a DNA-based method of species delimitation. Using a recent software (PhyloType), we explored evolutionary transitions in host-plant genera, feeding sites and geographic distributions in the diversification of *Cinara* and investigated how transitions in these characters have accompanied speciation events.

**Results:**

The diversification of *Cinara* has been constrained by host fidelity to conifer genera sometimes followed by sequential colonization onto different host species and by feeding-site specialisation. Nevertheless, our analyses suggest that, at the most, only half of the speciation events were accompanied by ecological niche shifts. The contribution of geographical isolation in the speciation process is clearly apparent in the occurrence of species from two continents in the same clades in relatively terminal positions in our phylogeny. Furthermore, in agreement with predictions from scenarios in which geographic isolation accounts for speciation events, geographic overlap between species increased significantly with time elapsed since their separation.

**Conclusions:**

The history of *Cinara* offers a different perspective on the mode of speciation of aphids than that provided by classic models such as the pea aphid. In this genus of aphids, the role of climate and landscape history has probably been as important as host-plant specialisation in having shaped present-day diversity.

## Introduction

Understanding processes that contribute to reproductive isolation and speciation is one of the most challenging areas in evolutionary biology. Allopatric speciation occurs when populations become spatially separated by regions of unsuitable habitat, which overcomes individual dispersal abilities and interrupts gene flow between those populations. Ecological speciation occurs when a transition in resource and/or habitat within ancestral species triggers gene flow interruption and the formation of new sister species [1,2]. This process can occur in allopatry (e.g. [3]) but is also supposed to permit sympatric speciation [4-6]. In the past decade, the role of ecological speciation in the diversification of phytophagous insects has been the focus of many studies and its importance compared to “ordinary” allopatric speciation has been reevaluated [4,7,8].

Host plants are considered as the main ecological factor involved in the speciation process of phytophagous insects. Several studies on various plant-feeding insects have clearly demonstrated the existence of host races and suggested that host based selection may underlie or at least speed up the speciation process in these organisms. Among textbook examples, are studies on *Rhagoletis pomonella* (Walsh) host races [5,9]. Aphids (Hemiptera, Aphididae) are also considered as model systems for the study of ecological speciation favored by adaptation to different host plants. These insects are often host specific and always mate on their host plants which make them good candidates for host driven speciation. There is much evidence for host shifts in the course of the evolution of aphids and examples of host plant specialisation events in several species have been reported (reviewed in [10]). The classic model for ecological speciation in aphids is the pea aphid (*Acyrthosiphon pisum* (Harris, 1776)) [11] in which incipient speciation seems to be in progress. Several studies have indeed shown that populations on different host plants (e.g. *Vicia* spp., *Trifolium* spp*.*, *Medicago* spp*.*) have diverged genetically and exhibit different stages of reproductive isolation [12-15]. Other studies have also shown that *Aphis gossypii* Glover, 1877 consists of several host-associated populations or races with a world-wide distribution [16,17].

All these studies suggest that host plant association is a major driver of reproductive isolation in aphids. However, explanations involving geographic isolation are seldom explored [10]. This might be because some aphid species are known to disperse over long distances, following aerial currents such as the jet stream, and successfully find their host plants during such events [18-20]. This characteristic could limit the influence of geographic barriers on gene flow. Furthermore, many aphid species are associated with economically important host plant species (crops, ornamental plants). Consequently, long range dispersal events associated with human transportation of infested plants are common. These result in many species exhibiting a very large geographic distribution (spreading over several continents): this very recent sympatry (on evolutionary time scale) might sometimes preclude investigating geographic isolation as a cause of species divergence while it might actually be relevant. Short distance dispersal, of 20 km or less, is probably usual in aphids [21] and the success of long range dispersion events is greatly dependent on the availability of host plants: aphids specialised on a few host species or host plants that have a restricted distribution are probably not successful in colonizing distant areas. Hence, allopatric speciation is a possible scenario for the diversification of aphids. Furthermore, host plants are not the only components of the ecological niche of aphids. Temporal shifts in life cycles [22] or changes in feeding habits on the plant can also be involved in reproductive isolation [23]. Hence, alternative scenarios to host-driven speciation probably deserve more attention than currently given in the aphid literature.

The conifer aphid genus *Cinara* Curtis, 1835 comprises 243 described species [24]. Approximately 154 occur in North America [25], 47 occur in Europe and the Mediterranean area [26] and about 40 occur in the Far East. *Cinara* species feed exclusively on the two conifer families that are found primarily in temperate and subtropical regions of the Northern hemisphere: Pinaceae and Cupressaceae. They exhibit a diversity of ecological features that make them good models to explore the importance of ecological specialisation in the diversification of aphids. Most species feed on a single or a few species of conifers, while others are less discriminatory, feeding on several species within a genus (e.g. *C. pinea* (Mordvilko, 1895) and *C. pergandei* (Wilson, 1919) on *Pinus* spp.) and sometimes even on several unrelated species of conifers (e.g. *C. confinis* (Koch, 1856) on *Abies* spp. and *Cedrus* spp.). Species of *Cinara* have specific feeding sites on their host plants. Some species feed only on young shoots, while others feed exclusively on large trunks [27]. However, some of their biological features also make *Cinara* species susceptible to geographic reproductive isolation. First, they have limited dispersal ability, compared to other aphid species. Their weight/wing length ratio is high [28] and some species are not recorded as producing winged morphs [25,29], which are the only ones able to disperse over large distances [19]. Then, because of their association with some conifer species living at higher altitude (some *Pinus, Picea* and. *Abies* spp.), some species are restricted to mountain ranges. Hence, many *Cinara* species show disjunct distributions and can encompass several allopatric populations [30].

Speciation processes can be investigated by phylogenetic studies and inferences about the evolution of characters (*e.g.*[31-33]). If speciation is only triggered by ecological changes, lineage splitting in the phylogeny should be accompanied by transitions in ecological characters and closely related species should not overlap in their ecological niche. Conversely, if speciation is not driven by ecological changes, the number of lineage splitting events in the phylogeny should be greater than the number of evolutionary transitions in ecological niche [32] and the phylogenetic reconstruction should reveal more niche conservatism than expected by chance. A phylogenetic study by Favret & Voetglin [30] on twenty-five species of North American *Cinara*, showed that closely related species used similar feeding sites on different host species. This study does not formally estimate the number of speciation events potentially triggered by ecological changes in *Cinara* but suggests that shifts in feeding sites followed by host plant specialisation may have driven the diversification of this group [30].

The objectives of this study were to reconstruct the phylogeny of European and North American *Cinara* species in order to investigate general trends in the diversification of this genus and test the scenario suggested by Favret & Voetglin [30] on a more global scale. More specifically, we aimed at estimating the relative importance of the two ecological characteristics (host plant species and feeding site) studied by Favret & Voetglin [30] versus geographic isolation in the speciation processes. We first investigated how geographic areas, host ranges and feeding sites were distributed along our phylogeny using the recently developed software PhyloType [34]. Given the distributions of characters across a set of taxa and a phylogeny, PhyloType determines if taxa sharing the same character states are more clustered than expected by chance on the phylogeny. If evolutionary transitions in host plant association and feeding sites were recurrently involved in the diversification of *Cinara*, sister taxa should diverge for these characters and we should not find any significant phylogenetic clusters associated with these characters. Conversely, the occurrence of strong geographic clusters on the phylogeny would be compatible with a scenario in which geographic isolation has not played a predominant role in the diversification of species within these clusters*.* We then followed the method of Nyman *et al.*[32] and reconstructed the evolution of a character summarizing the ecological niche (host plants species + feeding site) of each species. We estimated the proportion of lineage splits accompanied by a shift in this character and also tested whether species sharing the same niche were more clustered than expected by chance on our phylogeny. Finally, to give a coarse estimate of the importance of allopatric speciation in *Cinara*, we plotted geographic overlap among sister clades as a function of time since their divergence. According to theory, if allopatric speciation is prevalent, species that have recently differentiated should not have overlapping geographic ranges, while they may become more sympatric as time since divergence increases and their geographic range expands [33,35,36]. Altogether these analyses should give insights into the relative role of geography and ecology in the diversification of this aphid group.

## Materials and methods

### Taxonomic sampling

In an effort to capture the breadth of the phylogenetic, ecological and geographical diversity of *Cinara* species, we sampled 246 colonies representing 56 species of the genus *Cinara* (see Additional file 1). We sampled in both *Cinara* subgenera (52 species in the subgenus *Cinara* that encompasses 231 species and 4 species in the subgenus *Cupressobium* that encompasses 12 species). This represented 24% of the known species diversity. However, about 40 *Cinara* species listed in Blackman & Eastop [29] are only known from their original description or/and are suspected to be synonyms of other species, which suggests that our sampling was actually close to 30% of *Cinara* species. Whenever possible, we sampled several colonies per species to check for intraspecific genetic variation (our sampling varied from 1 to 18 colonies per species with an average of 4 colonies per species). Our sampling was focused on the western United States, which encompasses a large part of the species diversity of the *Cinara* genus, and France which with 26 species encompasses more than half of the European and Mediterranean *Cinara* fauna. Altogether we sampled in five states in the U.S., throughout six regions in France, and also sampled some specimens from Italy, Greece, Algeria and Kazakhstan. For each oligophagous species, we tried to sample colonies from different host species, in order to investigate the impact of host association on species differentiation, as well as from across their distribution range. Outgroup specimens were collected within the Lachninae subfamily in several genera (*Trama*, *Lachnus*, *Tuberolachnus*). Each colony was given a unique number and was geo-referenced. Host trees were identified to conifer genus and species when possible using local flora. Feeding sites, coloration and patterning in vivo were recorded for each colony and photographs were taken. As a destructive DNA extraction protocol was used, we selected vouchers among specimens from the same colony (*i.e.* sampled on the same host-plant at the same time) as the individual taken for extraction. Voucher specimens were mounted on microscope slides and deposited in the Aphididae collection of the Center for Biology and Management of Populations (CBGP) at Montferrier-sur-Lez, France. All specimens were identified by ACDA using mainly the keys of Blackman & Eastop [29] and Favret & Voetglin [37]. Collection details, host plant associations and nutrition sites as recorded in the field are given in Additional file 1.

### DNA extraction and sequencing

Total genomic DNA was extracted from a single individual per sample with the DNeasy Blood & Tissue Kit (Qiagen) in 120 μl of extraction buffer. We amplified several DNA fragments, two mitochondrial genes [the barcoding gene region (*cytochrome oxidase subunit I*: *COI*) and a fragment of the *cytochrome b* gene (*Cytb*)], two aphid nuclear DNA fragments [an approximately 770 bp intron corresponding to the para-type gene encoding the IIS2-S6 region of the voltage-gated sodium channel: *Aph,* and a portion of Elongation Factor (*EF*)], and two *Buchnera aphidicola* DNA fragments (*GroEL*: an approximately 550 bp fragment corresponding to a portion of the GroEL gene, a chaperonin assisting in the folding of proteins and *His*: an approximately 550 bp fragment of the ATP phosphoribosyltransferase (HisG) gene and histidinol dehydrogenase (HisD) gene and intergenic region). *Buchnera aphidicola* is the primary endosymbiont of aphids, which is transmitted from mother to offspring. It has been shown to cospeciate with its aphid hosts at several taxonomic levels in many groups of aphids and its genome has been used with success to reconstruct deep and shallow phylogenetic relationships in aphids [38-40]. Polymerase chain reactions (PCR) primers (Additional file 2) were designed for *Buchnera* DNA fragments using published genomes of *Buchnera aphidicola* in Genbank. Primer fidelity across taxa was not always consistent in *Cytb*, *His* and *GroEL*, we therefore defined several sets of primers for these DNA fragments. Despite our efforts, some specimens have slightly truncated sequence lengths.

PCR were performed in a final volume of 30 μl containing 1× reaction buffer (CoralLoad PCR Buffer, Qiagen), 0.1 mM of each dNTP, 0.7 μM of each primer, 1 U of *Taq* DNA polymerase and 1 μl of DNA extract. Sequencing reactions were carried out by MWG Operon, (Ebersberg, Germany) using the same primers as for PCR.

### Phylogenetic analyses

Sequences were aligned using ClustalW. Alignments of *COI*, *Cytb*, *His* and *GroEL* were straightforward due to a lack of length variation. *Aph* and *EF* comprised intergenic regions with indels, which complicated alignment between distantly related species. To avoid discarding information relevant for resolving shallower nodes of the phylogeny, we first aligned sequences of specimens for which intergenic regions were unambiguously aligned. All sequences were then aligned in one file by inserting gaps in ambiguously aligned regions in sequences that differed too much. This resulted in sequences having blocks of gaps aligned with intergenic regions of specimens that were too phylogenetically distant to assess site homology with confidence.

The alignment resulting from the concatenation of all DNA fragments is given in Additional file 3.

Alignments of the protein coding genes were translated into amino acids using Mega 4.0.2 [41] to detect frameshift mutations and premature stop codons, which may indicate the presence of pseudogenes (i.e. a fragment of nucleotide sequence that resembles a known protein’s domains but with stop codons or frameshifts mid-domain).

Phylogenetic trees were estimated using maximum likelihood (ML) and Bayesian methods. We first conducted ML searches on each DNA fragment. We checked for topological congruence between the trees and then combined all DNA fragments in a single DNA matrix. All analyses were conducted on a 150-core Linux Cluster at CBGP as well as on the CIPRES Science Gateway [42]. The data were partitioned into mitochondrial, nuclear and bacterial gene regions following [38]. The model with best fit for each partition was identified using the Akaike information criterion as implemented in MrAIC.pl 1.4.3 [43]. We performed ML analyses and associated bootstrapping using the MPI-parallelized RAxML 7.2.8-ALPHA [44]. GTRCAT approximation of models was used for ML bootstrapping [44] (1000 replicates). Bootstrap percentage (BP) > 95% was considered as strong support and a BP < 70% as weak support.

Bayesian analyses were conducted using a parallel version of MrBayes v. 3.2.1 [45]. We assumed across-partition heterogeneity in model parameters by unlinking parameters across partitions. Parameter values for the model were initiated with default uniform priors and branch lengths were estimated using default exponential priors. To improve mixing of the cold chain and avoid it converging on local optima, we used Metropolis-coupled Markov chain Monte Carlo (MCMC), with each run including a cold chain and three incrementally heated chains. The heating parameter was set to 0.02 in order to allow swap frequencies from 20% to 70% [46]. We ran two independent runs of 20 million generations. All values were sampled every 2000 generations. For the initial determination of burn-in, we examined the plot of overall model likelihood against generation number to find the point where the likelihood started to fluctuate around a constant value. Convergence was also evaluated using Tracer v1.5 [47]. The first 25% samples from the cold chains were discarded as burn-in. The results were based on the pooled samples from the stationary phases of the two independent runs. Posterior probabilities (PP) > 0.95 were considered as strong support and PP < 0.80 were considered as weak.

### Species delimitation

Several studies suggest that species delimitation is sometimes ambiguous within closely related species of aphids for two reasons. First, aphids are often identified based on host association, though such taxonomic treatment is correct only if aphids show a strict specialisation toward their host plants [48]. Second, aphid morphology often shows convergent evolution. *Cinara* species are no exception. The study by Favret & Voegtlin [30] and Foottit *et al.*[49] revealed several ambiguities in species delimitation with some mismatches between morphological species and genetic clusters. In our analyses, some species appeared divided into two or several phylogenetic clusters whose level of genetic divergence mirrored usual inter-specific level of genetic distance. To overcome these taxonomic issues, we used the species delimitation method of Pons *et al.*[50] to identify relevant entities for our study, i.e. genetic clusters of specimens potentially subject to selection and genetic drift.

The method of Pons *et al.*[50] identifies clusters representing independently evolving entities using a generalized mixed Yule coalescent model (GMYC). The model optimizes the maximum likelihood value of a threshold, such that the nodes before the threshold are identified as species diversification events, while the branches beyond the threshold are clusters following coalescent processes. Two ultrametric trees were constructed from the combined dataset using the uncorrelated lognormal relaxed clock method implemented in BEAST v1.7.4 [47], assuming either a Yule tree prior or a coalescent tree prior with a constant population size back through time. The same modelling strategies as for MrBayes and RAxML were used and clock models for each partition were unlinked. The relative times of divergence events were estimated by fixing the mean rate of molecular clock model to 1.0.

Two runs of 60 million generations with sampling every 6,000 generations were performed for the analysis assuming a coalescent tree prior. Two runs of 100 million generations with sampling every 10,000 generations were performed for the analysis assuming a Yule tree prior. For both analyses, the two separate runs were then combined using LogCombiner 1.7.4. We checked for convergence using Tracer 1.5 [47]. BEAST was also used to compare the goodness of fit of the two models based on Bayes factors (BF) [51,52] computed from harmonic mean estimators (HME) of the marginal likelihoods (1000 bootstrap replicates) as well as on the Akaike’s information criterion through MCMC (AICM). AICM has been shown to perform better in model selection than HME [53].

Following the removal of 10% burn-in, the sampled posterior trees were summarized using TreeAnnotator 1.7.4 to generate a maximum clade credibility (MCC) tree. The GMYC method as implemented in the R package SPLITS (http://www.rforge.r-project.org/projects/splits/), was then applied to the MCC tree that best fitted our data.

We then derived a “phylogenetic species” tree based on the results of the species delimitation method, by picking (at random) one specimen for each putative species and simply pruning subsequent specimens from the global tree with R using the package APE [54].

### Character analyses

We first annotated our specimens with sampling regions (states, province, and country), host plant (conifer genera and species when available) and feeding site(s) (trunk, branches, shoots) as recorded in the field. In a few cases, aphids were obtained by beating the branches of a tree, hence feeding sites could not be recorded for those samples. Character states attributed to each specimen are detailed in Additional file 1.

We then assigned a character state (for continent, host plant genus, host plant species and feeding site) to each cluster defined by the species delimitation method. To do so, we combined information recorded from the field for all specimens assigned to a species cluster with information available for each recognized species of *Cinara* compiled in the book by Blackman & Eastop [29] (updated in http://www.aphidsonworldsplants.info) and in [55] for species found in North America. Geographic areas were categorized into three character states: Nearctic, Palearctic and cosmopolitan species (the geographic origin of two cosmopolitan species is unknown). Host plant genera were split into seven character states: *Picea*, *Pinus*, *Abies*, *Larix*, *Cedrus*, *Pseudotsuga* (and occasionally *Abies*) and Cupressaceae as *Cinara* associated with this family often occurred on plants belonging to different genera (mostly *Cupressus* and *Juniperus*). Feeding sites were split into three categories: shoot, branch and trunk. When aphids assigned to a species cluster were found on non-lignified wood only (shoots, young twigs or at the base of new cones) in the field and according to information for the corresponding morphological species available in [29], they were considered as shoot feeders. When they were found capable of feeding on lignified wood (specimens were found on branches, older twigs and small trunks), they were considered as branch feeders. When they were found on trunks only, meaning that they needed a rostrum long enough to reach the sap through thick bark, they were considered as trunk feeders.

Information in Blackman & Eastop [29] is sometimes based on original descriptions or a few taxonomic surveys, therefore adding information from our field campaign generally increased species polymorphism in feeding sites and host plant use. Further, when a morphological species was divided into several clusters, we only took into account information recorded in the field in order to evaluate whether each cluster was associated with a particular character state.

We then used the PhyloType software [34] to explore the evolutionary trajectory of host plant genus association and feeding sites, and to give a coarse biogeographic scenario for the diversification of North American and European species. The PhyloType method can be summarized as follows (see [34] for details):

1) ancestral character state reconstruction using parsimony (ACCTRAN and DELTRAN algorithms).

2) identification of subsets of taxa having close phylogenetic relationships and common character states. Such subsets of taxa are called potential phylotypes. A phylotype has a unique character state at its root. All taxa in a phylotype share the same character state all along the path from every taxon included in the phylotype to the root of the phylotype.

3) identification of relevant phylotypes from among all potential phylotypes with combinatorial and numerical criteria. The choice of criteria and selection thresholds is left to users. Nevertheless, some constraints are imposed to avoid meaningless analyses. For instance, the *Size* criterion, which checks for a minimal number of taxa in the potential phylotype, is mandatory. Then come, the *Different* criterion which checks for a maximum number of sub-clades within the phylotype with different ancestral character states from that of the phylotype’s root and *Persistence*, which measures the extent to which the root character state of the phylotype is conserved in its descendants.

4) once phylotypes have been identified with the selected criteria, their significance can be assessed: character states are shuffled among the branches of the tree and the search for phylotypes is reiterated with the same criteria. The *p* values correspond to the fraction of shuffled data sets in which one finds a phylotype with the character being investigated and at least as large a size as the observed phylotypes.

PhyloType can therefore test whether characters are phylogenetically conserved and depict their evolution along the phylogeny.

We mapped the evolution of host plant genera, feeding sites and ancestral areas on our species phylogeny and determined if some significant phylotypes were associated with certain character states. The rationales behind conducting these analyses were to infer the number of transitions for each character and investigate how phylogenetically conserved they were; a character that is conserved is not the main driver of speciation. We also combined some of the characters and described their sequence of evolution throughout the diversification of the *Cinara* genus. For all analyses, criteria chosen for phylotype selection were as follows: size = 3, size/different=1, persistence=1, only nodes which ML bootstrap values were > 80 were taken into account in the analyses. Both ACCTRAN and DELTRAN optimization were tested and outgroups were excluded from the analyses. Shuffling procedures were performed with 1000 iterations.

We then estimated the proportion of lineage splits accompanied by a shift in resource use. We followed the procedure of Nyman *et al.*[32] and first identified all distinct ecological niches for the species included in our phylogeny (an ecological niche being defined by the combination of host plant species range and feeding sites). Each niche was coded with a different number. In doing so, we assumed that species ecological niches were overlapping when species shared one or several host plant species plus their feeding sites. Hence species were given a different niche number when there was no simultaneous overlap in host plant species and feeding sites with another species. We had three instances where one species shared part of its ecological niche with a first species and another part of its ecological niche with a second species; we either attributed two niche numbers to those or when these species overlapped in their niche with a sister species, we gave them the number corresponding to the niche of this sister species. Niche evolution was then optimized on the phylogenetic tree using maximum parsimony as implemented in PhyloType and we tested whether significant phylotypes were associated with these numbers (using the same procedure as described above for geography, host genus, and feeding sites). Furthermore, we also compared the parsimony score (number of steps) of the character “ecological niche” to the distribution of parsimony scores obtained from the 1000 shuffling made with PhyloType. This allowed for testing, given a tree and given the distribution of ecological niches across taxa, whether the total number of transitions in ecological niche was lower or higher than expected by chance.

To give a coarse estimate of the prevalence of allopatric speciation in *Cinara*, we plotted geographic overlap among sister species/ clades as a function of relative time since their divergence [33,35,36].

We indicated species geographic localization at a regional scale on our species phylogeny. Again, we compiled information from the literature [29] and our field campaigns to define eight geographic zones: North America (meaning the whole North American continent), Western North America (meaning all western states, from the North west coast of Canada to New Mexico), West of the Rockies (meaning the West coast of the US: California, Oregon, Washington), East of the Rockies (meaning in our sampling places in Colorado and New Mexico located East of the Rocky mountains), Europe (any country on the European continent), Mediterranean area (Algeria, Italy, Greece and the south East of France), Central Asia (Kazakhstan in our sampling), Cosmopolitan (the species were found worldwide). For documented cosmopolitan species that have recently spread over a continent, we used their area of origin. When a morphological species was divided into several clusters, we only took into account information recorded in the field in order to evaluate whether each cluster was restricted to a particular geographic zone.

For each node of the phylogeny, the overlap in present day geographic range of each clade/ species splitting at this node was set to 0 when no overlap existed between their geographic range and 1 when their geographic range overlapped. This variable was plotted against the depth of the node (i.e. the corresponding branch length in the ultrametric species tree). A logistic regression using this binary variable as a response variable and time as a factor was then calculated with R (using AOD and GGPlot packages). The prediction under a scenario where geographic speciation was prevalent was that the probability of overlap should increase with time [33].

## Results

### Sequence data

The final matrix contained 56 morphological ingroup species (five of those could not be assigned to either of two morphologically similar species, we thus gave them “mixed” names), six specimens for which identification keys did not lead to any known species and four outgroups, representing a total of 246 individuals and 4076 bp (*COI* + *Cytb* = 1418 bp, *Aph* = 257 bp, *EF* = 1135 bp, *His* = 719 bp, and *GroEL* = 547 bp). All sequences have been submitted to Genbank (Additional file 1). 1906 bp were variable and 1705 bp were parsimony informative. Sequences were missing for less than 12% of specimens for each DNA marker (18 *COI*, 5 *Cytb*, 8 *Aph*, 28 *EF*, 20 *His* and 25 *GroEl*)*.* Alignment of protein coding genes revealed no stop codons or frame shifts.

### Phylogenetic analyses

Models chosen by MrAIC for each partition were as follows: GTR + Γ (nuclear)*,* GTR + I + Γ (mitochondrial and *Buchnera aphidicola* fragments). Visual inspection of ML phylogenetic trees obtained with each independent partition showed no strong incongruences validating the use of combined analyses. Given that α and the proportion of invariable sites cannot be optimized independently from each others and following the recommendations provided in the RAxML manual, we used GTR + Γ with 4 discrete rate categories for all partitions.

ML and Bayesian analyses produced similar topologies. We obtained well-resolved phylogenetic trees (Additional files 4 and 5), in which most nodes were supported by high ML bootstraps and Bayesian posterior probability values.

### Species delimitation analyses

For each partition of the two analyses (assuming either a Yule tree prior or a coalescent tree prior), BEAST returned a 95% credible interval for the coefficient of variation of rates that was not abutting against zero, suggesting among branch rate heterogeneity (i.e., rejection of the molecular clock) [47]. Furthermore, the covariance statistics showed no strong evidence of autocorrelation of rates in the two combined phylogenies (covariance values spanning zero).

Bayes Factors (BF) and AICM both indicated that a Yule tree prior was a better fit to our data (log BF = 9.0; ∆AICM = −38.5). We therefore chose to use the topology obtained with a Yule prior to conduct the species delimitation method.

**Figure 1:**
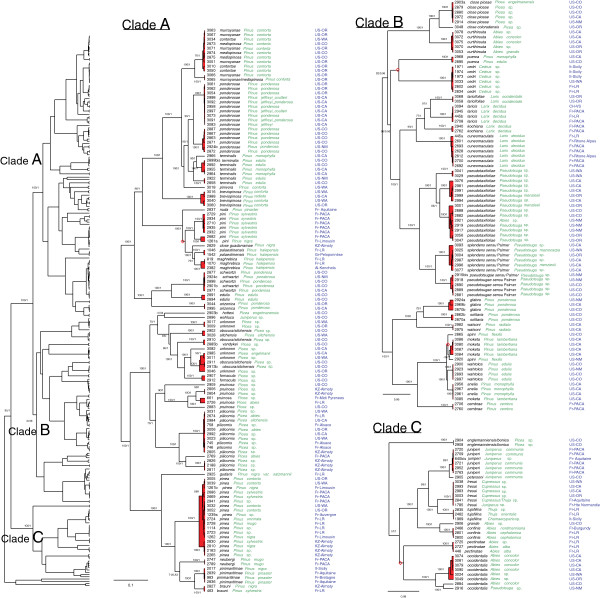
**Molecular phylogenetic hypothesis for *Cinara *species resulting from BEAST analysis using a Yule tree prior.** The topology on the left corresponds to the topology obtained with all specimens, it is divided into 3 sub-trees **A**, **B** and **C**, corresponding to the clades indicated in the global tree. Numbers at nodes correspond to ML bootstrap values > 50 and to BEAST posterior probability values > 0.80. **♦** Red rhombuses correspond to nodes that were not present in the ML tree. For each specimen, voucher number, species morphological identification, host species, sampling locality are indicated from left to right. The red coloration delimits clusters of specimens recognized as species by the GMYC method.

For both tree priors, MCC topologies were very similar to ML and Bayes topologies. We mapped node support values (*pp* and BP) obtained with ML and Bayesian analyses on the MCC topology obtained with a Yule tree prior (Figure 1).

The GMYC model was preferred over the null model of uniform (coalescent) branching rates (P < 0.001). Using the single-threshold GMYC model, 76 (CI = 70-94) putative species (54 genetic clusters and 22 singletons) were inferred for the MCC tree reconstructed using a Yule prior (T = −0.0097 substitutions/site). We further used the 76 putative species (72 ingroups species and four outgroups) inferred by the single-threshold GMYC model (Figure 1).

In most cases, phylogenetic species inferred by the GMYC model matched morphological species. However, we observed several mismatches. Some morphological species were clearly separated into two or even three genetically differentiated clusters. Among species associated with the genus *Pinus*, *Cinara ponderosae* (Williams, 1911) specimens formed two clusters, *C. terminalis* (Gillette & Palmer, 1924) specimens were split into three clusters, and *C. pini* (Linnaeus, 1758) specimens were split into two clusters. Among species associated with *Picea*, *C. pilicornis* (Hartig, 1841) was divided into three clusters and *C. pruinosa* (Hartig, 1841) into four clusters. Among species associated with *Abies* and/or *Pseudotsuga*, *C. pseudotaxifoliae* Palmer, 1952 formed three clusters and *C. occidentalis* (Davidson, 1909) formed four clusters.

In a few cases, our phylogenetic analyses revealed that some specimens were probably misidentified: one specimen (3085) associated with *Pinus lambertiana* Douglas, 1927 and identified as *Cinara moketa* Hottes, 1957 clustered with *Cinara anelia* (Favret & Voegtlin, 2004) (Figure 1, Clade B). The latter was known as being associated with *P. monophylla* Torrey et Frémont 1845 only while *C. moketa* was referenced as being associated with *P. lambertiana*. Our results therefore suggest that *C. anelia* might occasionally infest *Pinus lambertiana.*

Groups of specimens associated with *Pinus contorta* Douglas ex Loudon 1838 did not match morphological species (Figure 1, Clade A): there was one cluster including specimens identified as *C. murrayanae* (Gillette & Palmer, 1924), *C. medispinosa* (Gillette & Palmer, 1929) and *C. contortae* Hottes, 1958, and a second cluster including specimens identified as *C. murrayanae* only. *C. brevispinosa* (Gillette & Palmer, 1924) on the other hand formed a monophyletic cluster that was retrieved by all phylogenetic analyses as well as by the species delimitation method.

### Character analyses

**Figure 2:**
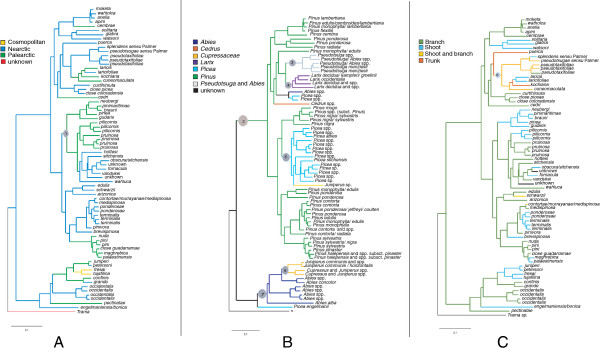
**Character history and phylotype identification as inferred by PhyloType (MP reconstruction, ACCTRAN optimization).** The topology used is from the ML analysis. Only significant phylotypes (whose sizes have a *p-*value ≤ 0.05) are indicated (filled circles at nodes with identifying numbers refer to phylotype identification in Table 1). **A)** Evolution of ancestral areas and phylotypes associated with this character, aphid species names are indicated as leaves of the tree. **B)** Evolution of host use and associated phylotypes, host species are indicated as leaves of the tree, N = not applicable. **C)** Evolution of feeding sites and associated phylotypes: aphid species names are indicated as leaves of the tree. Branches are coloured according to MP reconstruction (legends on the figure), and the root node identifiers of phylotypes are provided and refer to Table 1.

**Figure 3:**
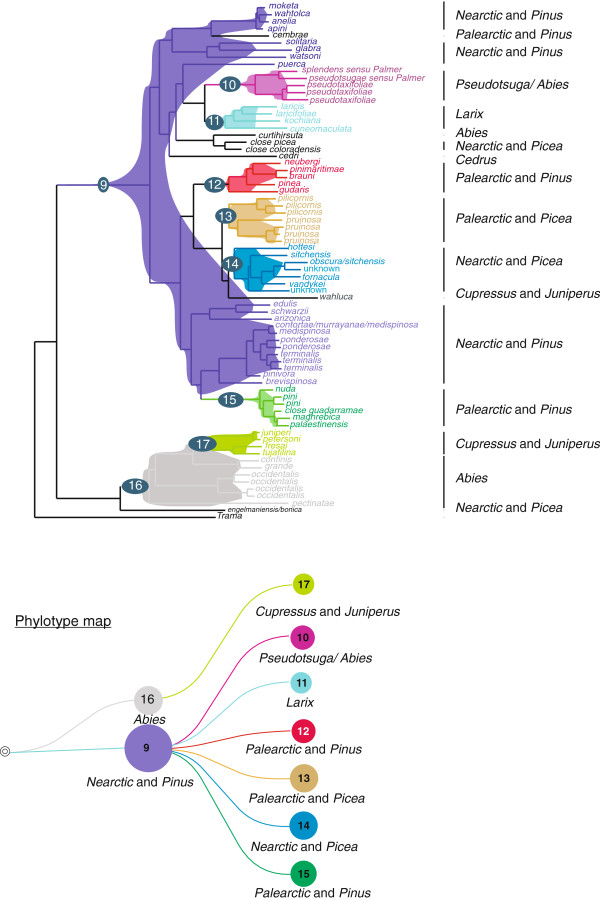
**Evolution of “Host range” combined with “Geographic range” and associated phylotypes.** The root node identifiers of phylotypes are provided and refer to numbers in Table 1, leave labels correspond to species names, the phylotype map below the tree summarizes the information in the tree.

Parsimony reconstructions as implemented in PhyloType suggested a Nearctic origin for *Cinara*, and several shifts to the Palearctic as well as one shift back to the Nearctic (14 transitions in character states for “geographic origin” when geography was defined as broadly as continents, Figure 2A). Using ACCTRAN optimization, a single significant geographic phylotype associated with the character state “Palearctic” was found (Table 1, Figure 2A, filled circles). Hence, lineage splitting events were often accompanied by geographic shifts. Conversely, the reconstruction of the history of host genera association revealed that this character was conserved in our phylogeny. The parsimonious reconstruction of the evolution of host range suggested only 10 evolutionary transitions for this character (Figure 2B). Species were clustered into 6 phylotypes corresponding to the 6 defined host ranges (Figure 2B). These phylotypes included 94% of the species in our phylogeny. This meant that there were very few lineage splits events associated with a shift to a new host genus. Reconstruction of the evolutionary trajectory of feeding sites yielded a single significant phylotype (Figure 2C, filled circle), meaning that there were many evolutionary transitions for this character. The parsimonious reconstruction of the evolution of feeding sites suggested 17 transitions (Figure 2C). No additional significant phylotype was found when combining “host plant genus” with “feeding sites” or “geographic origin” with “feeding site” (e.g. looking for phylotype “feeding on shoots of *Pinus*” or “feeding on trunks in the Nearctic”). However, combining the two main host plant genera (*Picea* and *Pinus*) with geographic origins, i.e. creating four new character states (*Picea* and Nearctic, *Picea* and Palearctic, *Pinus* and Nearctic, *Pinus* and Palearctic) yielded four significant additional phylotypes (Figure 3, Table 1), meaning that there was some geographic structure within species associated with *Pinus* on the one hand and with *Picea* on the other hand. Based on our results, the diversification of the genus can be summarized by Figure 3. *Cinara* associated with the genus *Abies* (P16 on Figure 3), indifferently of their geographic origin, formed a group of closely related species from which all species associated with Cupressaceae (P17) but one were derived. This latter phylotype (P17) actually corresponded to the subgenus *Cinara* group *Cupressobium*. A Nearctic species collected on *Picea*, which we failed to identify as either *C. engelmanniensis* (Gillette & Palmer, 1925) or *C. bonica* Hottes, 1956 (N° 2908), was found as sister species to the rest of this clade. Further diversification of *Cinara* stemmed from species associated with the genus *Pinus* in North America (P9). Within this latter phylotype, were nested two phylotypes associated with *Pinus* in Europe (P12, P15), one clade associated with the genus *Picea*, the latter being divided into a Nearctic phylotype (P14) and a Palearctic phylotype (P13). One North American species, *C. wahluca* Hottes, 1952 associated with a number of *Juniperu*s species was found as sister to this clade. Species on *Larix* and species on *Pseudotsuga* (and occasionally *Abies*) formed two separate phylotypes (respectively P11 and P10) with no geographic substructure and also derived from P9. Several singleton species were scattered in the phylogeny, namely *C. cedri* Mimeur, 1936 (a cosmopolitan species, native to North Africa and the South East Mediterranean region, associated with the genus *Cedrus*), *C. curtihirsuta* Hottes & Essig, 1954 a species associated with *Abies*, *C. cembrae* (Seitner, 1936) a European species associated with pine trees, and two unidentified North American species associated with *Picea*.

**Table 1:** Detailed table of the significant phylotypes found

**Analysis**	**P-label**	**Character state**	**Cov (%)**	**Sz**	**Ps**	**Df**	**Sz/Df**
Geographic origin (Figure 2A)	1	Palearctic	46	12, P = 0.004	2	1	12
Tot cov = 16%							
Host genus (Figure 2B)	2	*Pinus*	100	32, P = 0.008	3	4	8
Tot cov = 90.4%	3	*Pseudotsuga*	100	5, P < 0.0001	2	0	∞
	4	*Larix*	100	4, P < 0.0001	1	0	∞
	5	*Picea*	82	15, P < 0.0001	2	1	14
	6	Cupressaceae	80	4, P = 0.004	2	0	∞
	7	*Abies*	88	7, P < 0.0001	1	1	7
Feeding site (Figure 2C)	8	Shoot & branch	67	4, P = 0.005	2	3	1.3
Tot cov = 5%							
Host genus & geographic origin (Figure 3)	9	Nearctic &*Pinus*	100	20, P < 0.0001	3	11	11
Tot cov = 82.2%	10	*Pseudotsuga/ Abies*	100	5, P < 0.0001	2	0	∞
	11	*Larix*	100	4, P < 0.0001	1	0	∞
	12	Palearctic &*Pinus*	*42*	5, P = 0.054	*1*	*0*	∞
	13	Palearctic &*Picea*	100	7, P < 0.0001	2	0	∞
	14	Nearctic &*Picea*	70	7, P < 0.0001	1	0	∞
	15	Palearctic &*Pinus*	50	6, P = 0.001	1	0	∞
	16	*Abies*	88	7, P < 0.0001	1	1	7
	17	Cupressaceae	80	4, P = 0.002	2	0	∞
Ecological niche (Figure 4)	18	Shoots and branches of *Pseudotsuga* spp. and/or *Abies* spp.	100	5, P < 0.0001	2	0	∞
Tot cov = 56.2%	19	Shoots of *Pinus* spp. subsect. *Pinus*	100	4, P < 0.0001	1	1	4
	20	Shoots of *Picea* spp.	60	3, P = 0.002	1	0	∞
	21	Branches of *Picea* spp.	70	7, P < 0.0001	1	5	1.4
	22	Branches of *Pinus contorta*	100	3, P = 0.002	1	2	1.5
	23	Shoots of *Pinus edulis* and/or *monophylla*	100	3, P = 0.003	1	0	∞
	24	Branches of *Pinus sylvestris* and/or *P. nigra*	100	3, P = 0.003	1	1	3
	25	Shoots of *Pinus* spp. subsection *Pinaster*	100	3, P < 0.0001	1	0	∞
	26	Shoots of *Juniperus* spp.	100	4, P < 0.0001	2	0	∞
	27	Branches of *Abies* spp.	86	6, P < 0.0001	2	1	2

The analyses were run with ACCTRAN option (similar results with DELTRAN option). Abbreviations are: P-label, identifier of phylotype; Cov, coverage, i.e. percentage of taxa annotated with Character state that belongs to the phylotype. The *p-*value of *Sz* is given in the corresponding column.

Selection criteria are *Size* (Sz ≥ 3), *Size*/*Different* (Df ≤ 1), *Persistence* (Ps ≥ 1) and *Support* (Sp ≥ 0.80).

**Figure 4:**
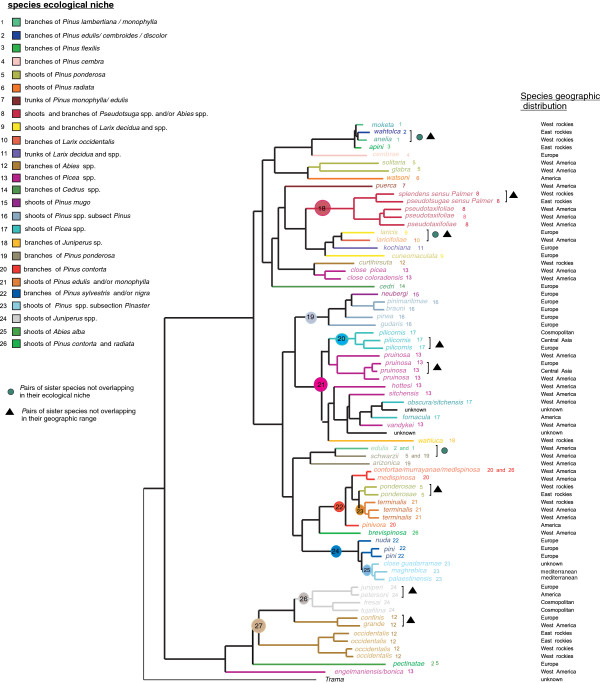
**Evolution of niche differentiation among species as inferred by MP using Phylotype: branch colors and numbers correspond to a niche (an ecological niche being defined by the combination of host-plant species range and feeding sites).** Species names are followed by a number referring to their ecological niche. The root node identifiers of phylotypes are provided and refer to numbers in Table 1. Fine scale (regional) geographic distribution of each species is indicated at the right end of the figure. ● Green dots indicate species pairs that do not overlap in their ecological niche. ∆ Black triangles indicate species pairs that do not overlap in their geographic distribution.

The existence of 72 ingroup species in our phylogenetic tree requires at least 71 past speciation events. The reconstruction of the history of ecological niches suggested that there have been 31 transitions for this character (Figure 4). This meant that among the 71 “speciation” events in our tree, less than half of them were accompanied by a shift in resource use. The results of 1000 shufflings, showed that the number of changes in ecological niche was lower than expected by chance (P < 0.001). Phylotype analyses yielded 10 significant phylotypes associated with ecological niche (Table 1) meaning that among species sharing the same ecological niche, there were 10 species groups that were more clustered than expected by chance. To give a different estimate of the importance of shifts in resource use in species differentiation, when only sister species on the ML species tree were considered, only three out of 21 pairs had non overlapping niches (i.e. 15%), and among those three, two had also non overlapping geographic ranges (Figure 4).

As a comparison, 8 out of these 21 pairs (38%) had non-overlapping geographic ranges (Figure 4). The results of the logistic regression showed that the probability of clades overlapping in their contemporary geographic range increased significantly with time since their divergence (Coeff =32.82, *P=*0.038) (Additional file 6), which followed the prediction of the scenario in which geographical isolation played a significant role in the speciation process.

## Discussion

### Molecular systematics of the genus Cinara

We obtained a well-resolved phylogeny of the genus *Cinara* encompassing a quarter of the species diversity. Our results confirmed the monophyly of the subgenus *Cupressobium* though it appeared nested within a group of species belonging to the subgenus *Cinara*. These relationships suggest that the subgeneric classification within *Cinara* needs revision. The data presented here will be useful for future work dealing with the taxonomy of the genus.

Our molecular work on species delimitation was largely concordant with previous taxonomic investigation in the genus [25,37,56,57]. Overall there was a good correspondence between morphological species and genetic clusters inferred by the GMYC species delimitation method. But our analyses revealed more diversity than currently described in some common species with large geographic distributions: *C. pilicornis*, *C. pruinosa*, *C. ponderosae*, *C. terminalis, C. pini* and *C. occidentalis*. The subdivision of *C. terminalis* into several lineages was already suggested by [30]. In addition, species clusters on *Pinus contorta* did not correspond to morphological identifications. These results confirmed that taxonomic relationships in this group of species require further investigation [29,58]. The published taxonomic treatments of the genus *Cinara* are quite extensive. This is probably because the genus encompasses several forest pests that are sometimes considered invasive [59,60]. Some species are important producers of forest-honey [61] and they show variations in their natural history, which make them attractive to biologists. The concordance of our molecular results with morphological taxonomy on our broad geographical sampling suggests that biological information (patterns of host-plant association, geographic distribution, feeding sites) compiled from the taxonomic literature are reliable and can be used with confidence to infer character history on phylogenetic trees.

### Distribution patterns across continents and among conifer genera

Groups of phytophagous insects with large Holarctic distributions offer good models for comparing the effects of host shifts and geographic events on their diversification [62]. We can investigate whether their phylogenetic history reflects host conservatism followed by range expansion and diversification with their host or geographic clustering of closely related species and opportunistic shifts to new unrelated hosts. Our reconstructions of the history of the genus *Cinara* and PhyloType inferences depict a global diversification scenario where speciation processes are strongly constrained by host genus association. There are few transitions in host plant genera that have occurred early in the diversification of the genus. Each *Cinara* clade associated with a particular host genus naturally present on both continents encompasses Nearctic and Palearctic *Cinara* species. In two of the most diverse Pinaceae genera (*Pinus* and *Picea*), geographic clustering of species at continental scales is observed. This pattern of distribution suggests repeated independent evolution of Nearctic and Palearctic lineages in each “host genus cluster” via large scale geographic isolation. The recurrent presence of species from two continents in relatively terminal positions on phylogenetic trees is not rare in phytophagous insects [62] and these patterns are always interpreted as reflecting speciation by geographic processes such as vicariance and dispersal (see [63] and [64] for studies on aphids).

Understanding the distribution of *Cinara* species at continental scales requires more thorough biogeographic analyses (*e.g.*[65]). This is beyond the scope of this paper as elaborating robust scenarios would require including Asian species (about 40 known species), species restricted to Eastern North America (about 10-15 species) and fossil data to calibrate the phylogeny. In any case, the genus *Cinara* probably offers a good model to test hypotheses on historical biogeography of the Holarctic fauna [66]. The coarse scenario given by our analyses already suggests that the diversity of the genus has probably arisen from the Nearctic zone. Numerous faunal exchanges between the Palearctic and Nearctic are then highlighted by our analyses. Inferring the relative importance of vicariance versus dispersal events to explain this pattern requires further study. It is noteworthy that many *Cinara* species are now found worldwide, which clearly reflects the impact of human activities on aphids’ dispersal. For instance, many species associated with *Cupressus* and *Thuja*, often used as ornamental hedges, are found across the globe. Some *Cinara* species (*C. pilicornis*, *C. pruinosa*) also probably hitchhike around the world with Christmas trees (several *Picea* spp. *and Abies* spp.). Therefore, inferring a proper biogeographical history for the genus will necessitate categorizing species that have been transported around the world relative to locations of natural populations.

Interestingly, the history of host genera association throughout the evolution of *Cinara* does not parallel conifer phylogeny [67-69]. In the classification of conifers, Cupressaceae and Pinaceae form two well-differentiated families. Within Pinaceae, *Abies*, *Tsuga* and *Cedrus* form a sister clade to the other genera hosting *Cinara*. Within this latter group, *Larix* + *Pseudotsuga* are sister to *Pinus* + *Picea*[67]. In our phylogeny, most *Cinara* species associated with Cupressaceae (P6 Figure 2B, and P17 Figure 3) have evolved from an *Abies* feeding ancestor: this represents a shift to a distant host. The colonisation of all Pinaceae genera then arose from *Cinara* associated with *Pinus* (P2 Figure 2B and P9 Figure 3). The only phylogenetic relationships in *Cinara* that mirrors conifer genera relatedness is the proximity of species feeding on *Larix* and *Pseudotsuga*. Therefore, the history of *Cinara* reflects shifts to available hosts that are not always closely related. Comparing this history with the biogeographic history of associated conifer genera will allow clarification of how constraints linked with host association and *Cinara*’s biogeographic history are entangled and have shaped present-day diversity.

Previous studies of conifer-feeding insects in the Holarctic region have also rejected the hypothesis of independent radiations of these insects in Europe, Asia and North America [70-72]. The lack of resolution and/or incomplete sampling in these phylogenies does not allow for a detailed comparison of the evolution of the association with conifer genera with our results. However in contrast to our study, the history of *Megastigmus* (Hymenoptera: Torymidae) [71] suggests that species on Cupressaceae form a distinct clade from species feeding on Pinaceae (including Abies-feeding species). On the other hand, the relatedness of species feeding on *Pseudotsuga* and *Larix* was also found in a *Dendroctonus* (Scolytidae) phylogeny [72,73]. Comparative historical biogeography of these conifer-feeding groups should provide insights on the role of conifer history on the diversification of phytophagous insects with similar ecological requirements in the Holarctic.

### *Speciation in the genus* Cinara

In the last decade, the topic of ecological speciation has fueled many debates in the literature [7] and stimulated many research projects, such as the search for signature of this process in various genomes [74-80]. Consequently, studying “ordinary” geographic speciation has almost become an unusual area of research. As Coyne and Orr [81] mention in their book on speciation “allopatric speciation appears so plausible that it hardly seems worth documenting”. Ecological speciation is definitely a plausible scenario for several taxa [82-84] and several studies have convincingly demonstrated that this process can occur in sympatry [85,86]. However, these studies were generally conducted on very closely related species or lineages that have not always achieved complete reproductive isolation [6,85,87]. Because of this narrow focus on a few model species, it is difficult to give an estimate of the frequency of ecological speciation even for species belonging to the same taxonomic groups as the focal species. This needs to be approached by broader macro-evolutionary studies. Phylogenetic studies on the patterns of diversification of phytophagous insects have largely focused on the role of host plant shifts in the speciation process (e.g. [88-94]). This also applies to aphids (see [10] for a review but see also [63,64,95] for other views). However, these studies rarely evaluate the frequency of speciation by host shifts or weight it against alternative modes of speciation.

Our analyses on the conifer-feeding aphid genus, *Cinara*, reveal a pattern of frequent niche shifts in terms of host plant use and feeding habits. *Cinara* species show frequent host specialisation events and multiple transitions from branch-feeding to shoot-feeding. We do not find the clustering of species using similar feeding sites observed by Favret and Voegtlin [30] on North American Pinyon pines *Cinara* on a broader scale. Despite these multiple transitions, mapping the evolution of ecological niche on our phylogeny shows that less than 50% of lineage splits have been accompanied by a shift in resource use. Though parsimony can tend to underestimate the frequency of changes [96], this should not affect our conclusions, as randomization tests clearly show that the probability of observing 31 changes is much lower than expected by chance. If we look more precisely at terminal nodes of our phylogeny, phylogenetic clusters revealed by the species delimitation method do not correspond to host specialised races or lineages specialised on particular feeding sites, and only a few sister species pairs differ in their resource use (15%). Given that there is a broad range of host specificity in *Cinara* (species range from strictly monophagous to highly polyphagous), this is not that surprising. Such diversity in feeding diets already suggests that different speciation mechanisms have been acting. Ecological speciation via host shifts in generalist lineages (e.g. feeding on several *Pinus* species or even across conifer families in our biological model) can only occur by shifts to higher plant taxa, i.e. by shifting to a new plant genus, which probably require important physiological and behavioural changes that might constitute rare events (see [97] for a thorough discussion on the importance of niche width in ecological speciation). Our estimate of the number of ecology driven speciation events is similar to the finding of Nyman *et al.*[32] on sawflies (Hymenoptera: Tenthredinidae), and the conclusions from a broad literature survey of macroevolutionnary studies by Winkler & Mitter [98] that both suggest that the ratio of ecological versus non-ecological speciation in phytophagous insect is at the most “1:1”. This also echoes the results of Imada *et al.*[99] on a group of 25 phytophagous moth species showing that none of them have speciated via ecological speciation and a study by Roesch Goodman *et al.*[100] that demonstrates that geography and not ecology is responsible for the diversification of host specific Hawaiian plant hoppers. Furthermore, as underlined by previous authors [31,35], showing that a shift in ecological resource has accompanied a speciation event does not mean that ecological differentiation has triggered the formation of two species. Resource shifts frequently occur after speciation. Therefore, our estimate of 50% of ecology-based speciation events is an upper limit. This result contrasts with the signature of geographical isolation imprinted in the biogeographical history of the genus, as outlined in the previous paragraph. The comparison of sister-species pairs (there are more sister species that do not overlap in their geographic range than species that do not overlap in their resource use) and the fact that geographic overlap between lineages tends to increase with time elapsed since the speciation event also indicate that geographic isolation has promoted speciation events in *Cinara*, Geographical barriers such as mountain ranges have probably shaped some of the diversity at the regional scale. Indeed, in some western North American species, we observe a disjunct distribution in sister species pairs, with some species being restricted on either side of the Rocky Mountains [for instance *Cinara splendens* (Gillette & Palmer, 1924) and *Cinara pseudotsugae* (Wilson, 1912) (Figure 4)]. *C. ponderosae* is also subdivided into two sub-clades (as identified by the species delimitation method) found either West or East of the Rockies (Figure 4). Western North America actually contains a large part of the diversity of *Cinara* (about 40% with more than 85 species [29]). Topographic complexity in western North America [101] and more specifically in the southern Rocky mountains and intermontane plateau has been suggested to be responsible for high diversification rates in several organisms including several insects [102,103]. It is also probably implicated in the diversification of *Cinara*. Evidence for geographical isolation linked with long distance is not always obvious in the terminal nodes of our phylogeny. If we compare Central Asian populations with European populations of several species exhibiting a large Palearctic distribution, they do not always show significant divergence. *C pinea* specimens from Europe and Central Asia appear divided into two separate clades, however these clades are not diverged enough to be recognized as different species (Figure 1, clade A). However, in *C. pilicornis*, a Kazakh cluster is retrieved that is clearly differentiated from the rest of the specimens (Figure 1, clade A). This east–west divergence can be interpreted as a result of range expansion across the Palearctic and subsequent isolation by distance. Nevertheless, within the European cluster, there is one Kazakh-type individual, which may correspond to a recent long dispersal event associated with human transport. American specimens from *C. pinea* and *C. pilicornis* are entangled within European specimens which agrees with the fact that they have been recently introduced (early last century) into North America from Europe [55].

Several issues could bias our conclusions. First our sampling only encompasses a third of the diversity of the genus and missing species might influence the results of our studies. However, our sampling was mainly focused on maximizing the number of species sampled from one geographical location rather than sampling the genus throughout its geographic range (for instance within Colorado (US) we have included 21 species among the 39 that are recorded from this state). The vast majority of species missing from our study is restricted to eastern Nearctic or occurs throughout Asia. Therefore, we believe that adding species in our analyses will add at least as much geographic variation as ecological variation and that it should not significantly affect the proportion of speciation events driven by ecological niche shifts inferred by our analyses. More importantly, taxonomic issues concerning host plants might have influenced our conclusions. Host identification can be difficult and *Pinus* sub-species or hybrids that we have not managed to identify might occur throughout our sampling. For instance, *Pinus ponderosa* and *P. lambertiana* encompass infra-specific diversity [104-106]. Some of the species diversity in *Cinara* attributed to geographic factors might actually reflect specialisation to particular hybrid or subspecies. In the *Picea* genus, many species are planted as ornamental trees and occur outside their geographic range, which renders identification quite difficult. *Cinara* feeding on *Picea* are often indicated as feeding on *Picea* spp. [29] with no precise definition of their host range. We have also found a lot of “phylogenetic species” within widely distributed species feeding on *Picea* (e.g. *C. pruinosa* and *C. pilicornis*). This suggests that the taxonomic treatment of *Cinara* species on *Picea* and determination of their host associations might have been less thorough than the treatment of species associated with *Pinus*. Hence, patterns of host association in *Picea*-feeding species in the literature and our study probably lack precision and we might have failed at identifying host specialisation events on *Picea* species. However, it is also likely that there have also been more vicariance events than suggested by our phylogeny. Present day distributions of many *Cinara* species are obviously the result of range expansion. Climatic history has probably contributed to repeated instances of range contraction and range expansion in response to glacial cycles. These changes in geographic distributions could have led to sympatry in many species of our study while they had actually separated in allopatric conditions. But there are actually many cases where geographic factors and hosts adaptation are simply confounded. The species diversity and intraspecific variation in *Cinara* hosts trees have also resulted from geographic events. Pine tree species occurring naturally in the Nearctic region are different from species occurring in Europe. Climatic fluctuations occurring during the last glacial cycles have also probably affected the genetic structure of *Cinara*’s host plants, especially in the mountainous environments of western North America where conifer diversity is high. Therefore, when the genetic clusters observed in aphids mirror the genetic structure of their hosts and also correspond to isolated geographic zones; it will be difficult to tell apart geographic isolation from host adaptation in the speciation process. Finally, we have limited our definition of ecology-based speciation events to events due to host shifts and/or changes in feeding sites. We believe that these are the two main ecological factors that could lead to species divergence in our system. However, divergence in the timing of reproduction [22] and changes in reproductive modes could also be driven by ecological forces such as competition or escape from parasites [107], and might account for some speciation events in *Cinara.*

## Conclusions

Our broad inference regarding diversification within *Cinara* suggests that even in this group of specialised phytophagous insects, ecological differentiation linked with host plant and feeding sites shifts is not the sole driver of speciation. In this aphid genus, climatic events and landscape history are probably as important as ecology in having shaped present day diversity. The history of *Cinara* offers a different view on the processes of speciation in aphids than that provided by models such as the pea aphid.

## Competing interests

The authors declare that they have no competing interests.

## Authors’ contributions

EJ designed the study with ACDA, participated in specimen sampling, sequence acquisition, alignment, character inference and drafted the MS. AC conducted phylogenetic analyses and participated in the writing of the paper. GG participated in DNA sequence acquisition and analyses. FC helped with tree illustration and the use of software. RF participated in the writing of the paper. ACDA conducted specimen sampling and all taxonomic identifications and participated in the writing of the paper. All authors read and approved the final manuscript.

## Supplementary Material

Additional file 1 Table with sample information and Genbank accession numbers. Click here for file

Additional file 2 Table with PCR primer information. Click here for file

Additional file 3 Nexus file used for phylogenetic analyses. Click here for file

Additional file 4 Phylogenetic topology obtained by ML analyses. Click here for file

Additional file 5 Phylogenetic tree obtained with BI. Click here for file

Additional file 6: Figure S1 Plot of species geographic overlap vs an estimate of their divergence time. Click here for file
