# Supplementary material for: Is ecological speciation a major trend in aphids? Insights from a molecular phylogeny of the conifer-feeding genus Cinara
Source: Front Zool. 2013 Sep 18;10:56. doi: 10.1186/1742-9994-10-56 (PMC3848992; doi:10.1186/1742-9994-10-56)
Supplement: Additional file 2 — Table with PCR primer information. [file 1742-9994-10-56-S2.doc]

Table S2: Names, sequences and references of primers used for PCR and sequencing.

| DNA fragment | Name of primer | Sequence of primer | References |
| --- | --- | --- | --- |
| COI | LepF | ATTCAACCAATCATAAAGATATTGG |  |
|  | LepR | TAAACTTCTGGATGTCCAAAAAATCA |  |
| Cytb | CP1 | GATGATGAAATTTTGGATC |  |
|  | CP2 | CTAATGCAATAACTCCTCC |  |
| EF | Ef3 | gaacgtgaacgtggtatcac |  |
|  | Ef6 | TGACCAGGGTGGTTCAATAC |  |
| Aph | Aph13 | | GATTGAATCAATGTGGGACTGCTTAC | | --- | |  |
|  | Aph15 | ACTCAGCAACAACGCCAAGAAAAG |  |
| His | HisF  HisF2 | | CDTTTKCTGAAAATATGCCWA  ATGATGATATHCCKGGWTTAATTATGGA | | --- | | This study |
|  | HisR  HisR2 | \| YGGTAAWACYAAAATRGAACTAGC  ATATATTTDGATTCTCKDGCTTTAAT \| \| --- \| | This study |
| Groel | groelintF  groelCinF2  groelCinf3 | | TTAATTATTTCAGAAGATTTAGAAGG  GGGTGCGCAAATGGTMAARGAATA  AGATTTAGAA GGWGARGCDTTAGC | | --- | | This study |
|  | groelRbis  groelRbis2 | \| CCAACRTTYTGATCTTCRTTTTSACC  CCAACATTTT GATCTTCRTTTTGACC \| \| --- \| | This study |

1. Hajibabaei M, Janzen DH, Burns JM, Hallwachs W, Hebert PDN: **DNA barcodes distinguish species of tropical Lepidoptera.** *PNAS* 2006, **103:**968-971.

2. Harry M, Solignac M, Lachaise D: **Molecular evidence for parallel evolution of adaptive syndromes in fig-breeding Lissocephala (Drosophilidae).** *Mol Phylogenet Evol* 1998, **9:**542-551.

3. von Dohlen CD, Kurosu U, Aoki S: **Phylogenetics and evolution of the eastern Asian-eastern North american disjunct aphid tribe, Hormaphidini (Hemiptera: Aphididae).** *Molecular Phylogenetics and Evolution* 2002, **23:**257-267.

4. Carletto J, Blin A, Vanlerberghe-Masutti F: **DNA-based discrimination between the sibling species *Aphis gossypii* Glover and Aphis frangulae Kaltenbach.** *Systematic Entomology* 2009, **34:**307-314.
