# Supplementary figures and images for: Is ecological speciation a major trend in aphids? Insights from a molecular phylogeny of the conifer-feeding genus Cinara
Source: Front Zool. 2013 Sep 18;10:56. doi: 10.1186/1742-9994-10-56 (PMC3848992; doi:10.1186/1742-9994-10-56)

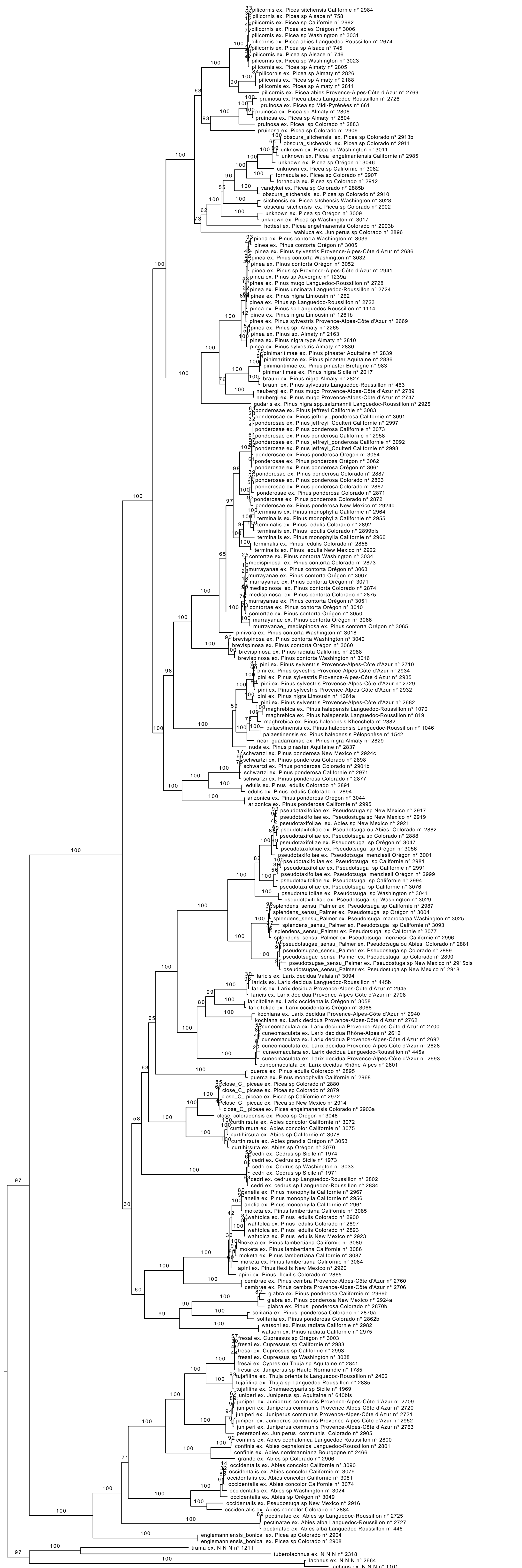

Supplement: Additional file 5 — Phylogenetic tree obtained with BI. [file 1742-9994-10-56-S5.pdf]
