# Supplementary material for: Is ecological speciation a major trend in aphids? Insights from a molecular phylogeny of the conifer-feeding genus Cinara
Source: Front Zool. 2013 Sep 18;10:56. doi: 10.1186/1742-9994-10-56 (PMC3848992; doi:10.1186/1742-9994-10-56)
Supplement: Additional file 6: Figure S1 — Plot of species geographic overlap vs an estimate of their divergence time. [file 1742-9994-10-56-S6.docx]

Figure S1: Plot of present -day geographic overlap (0= no overlap, 1= overlap) of clades, splitting at each node of the tree, function of time since the divergence of the clades (depth of the node in the ultrametric phylogenetic tree). The logistic regression curve is plotted on the graph.

Results of the Logistic regression:

Coefficients:

Estimate Std. Error z value Pr(>|z|)

(Intercept) 0.3137 0.6480 0.484 0.6283

Node_depth 32.8264 15.8266 2.074 0.0381 *
