# Supplementary material for: Is ecological speciation a major trend in aphids? Insights from a molecular phylogeny of the conifer-feeding genus Cinara
Source: Front Zool. 2013 Sep 18;10:56. doi: 10.1186/1742-9994-10-56 (PMC3848992; doi:10.1186/1742-9994-10-56)
Supplement: Additional file 4 — Phylogenetic topology obtained by ML analyses. [file 1742-9994-10-56-S4.pdf]

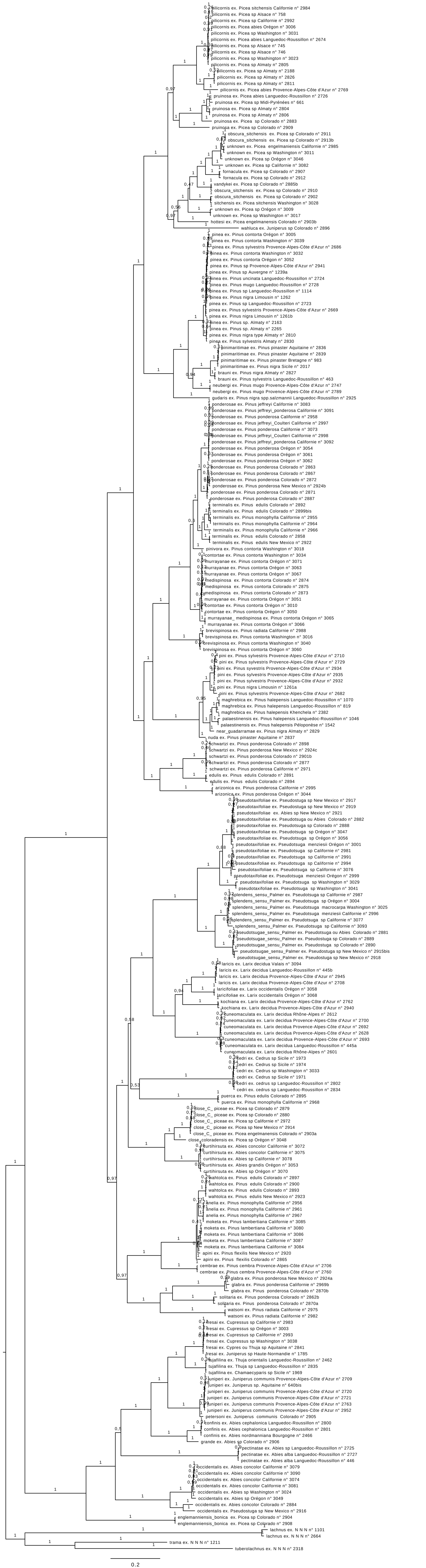

pilicornis ex. Picea sitchensis Californie n° 2984
pilicornis ex. Picea sp Alsace n° 758
pilicornis ex. Picea sp Californie n° 2992
pilicornis ex. Picea abies Orégon n° 3006
pilicornis ex. Picea sp Washington n° 3031
pilicornis ex. Picea abies Languedoc-Roussillon n° 2674
pilicornis ex. Picea sp Alsace n° 745
pilicornis ex. Picea sp Alsace n° 746
pilicornis ex. Picea sp Washington n° 3023
pilicornis ex. Picea sp Almaty n° 2805
pilicornis ex. Picea sp Almaty n° 2188
pilicornis ex. Picea sp Almaty n° 2826
pilicornis ex. Picea sp Almaty n° 2811
pilicornis ex. Picea abies Provence-Alpes-Côte-d'Azur n° 2769
pruinosa ex. Picea abies Languedoc-Roussillon n° 2726
pruinosa ex. Picea sp Midi-Pyrénées n° 661
pruinosa ex. Picea sp Almaty n° 2804
pruinosa ex. Picea sp Almaty n° 2806
pruinosa ex. Picea sp Colorado n° 2883
pruinosa ex. Picea sp Colorado n° 2909
obscura_sitchensis ex. Picea sp Colorado n° 2911
obscura_sitchensis ex. Picea sp Colorado n° 2913b
unknown ex. Picea engelmaniensis Californie n° 2985
unknown ex. Picea sp Washington n° 3011
unknown ex. Picea sp Orégon n° 3046
unknown ex. Picea sp Californie n° 3082
fornacula ex. Picea sp Colorado n° 2907
fornacula ex. Picea sp Colorado n° 2912
vandykei ex. Picea sp Colorado n° 2885b
obscura_sitchensis ex. Picea sp Colorado n° 2910
obscura_sitchensis ex. Picea sp Colorado n° 2902
sitchensis ex. Picea sitchensis Washington n° 3028
unknown ex. Picea sp Orégon n° 3009
unknown ex. Picea sp Washington n° 3017
hottesi ex. Picea engelmanensis Colorado n° 2903b
wahluca ex. Juniperus sp Colorado n° 2896
pinea ex. Pinus contorta Orégon n° 3005
pinea ex. Pinus contorta Washington n° 3039
pinea ex. Pinus sylvestris Provence-Alpes-Côte d'Azur n° 2686
pinea ex. Pinus contorta Washington n° 3032
pinea ex. Pinus contorta Orégon n° 3052
pinea ex. Pinus sp Provence-Alpes-Côte d'Azur n° 2941
pinea ex. Pinus sp Auvergne n° 1239a
pinea ex. Pinus uncinata Languedoc-Roussillon n° 2724
pinea ex. Pinus mugo Languedoc-Roussillon n° 2728
pinea ex. Pinus sp Languedoc-Roussillon n° 1114
pinea ex. Pinus nigra Limousin n° 1262
pinea ex. Pinus sp Languedoc-Roussillon n° 2723
pinea ex. Pinus sylvestris Provence-Alpes-Côte d'Azur n° 2669
pinea ex. Pinus nigra Limousin n° 1261b
pinea ex. Pinus sp. Almaty n° 2163
pinea ex. Pinus sp. Almaty n° 2265
pinea ex. Pinus nigra type Almaty n° 2810
pinea ex. Pinus sylvestris Almaty n° 2830
pinimaritimae ex. Pinus pinaster Aquitaine n° 2836
pinimaritimae ex. Pinus pinaster Aquitaine n° 2839
pinimaritimae ex. Pinus pinaster Bretagne n° 983
pinimaritimae ex. Pinus nigra Sicile n° 2017
brauni ex. Pinus nigra Almaty n° 2827
brauni ex. Pinus sylvestris Languedoc-Roussillon n° 463
neubergi ex. Pinus mugo Provence-Alpes-Côte d'Azur n° 2747
neubergi ex. Pinus mugo Provence-Alpes-Côte d'Azur n° 2789
gudaris ex. Pinus nigra spp.salzmannii Languedoc-Roussillon n° 2925
ponderosae ex. Pinus jeffreyi Californie n° 3083
ponderosae ex. Pinus jeffreyi_ponderosa Californie n° 3091
ponderosae ex. Pinus ponderosa Californie n° 2958
ponderosae ex. Pinus jeffreyi_Coulteri Californie n° 2997
ponderosae ex. Pinus ponderosa Californie n° 3073
ponderosae ex. Pinus jeffreyi_Coulteri Californie n° 2998
ponderosae ex. Pinus jeffreyi_ponderosa Californie n° 3092
ponderosae ex. Pinus ponderosa Orégon n° 3054
ponderosae ex. Pinus ponderosa Orégon n° 3061
ponderosae ex. Pinus ponderosa Orégon n° 3062
ponderosae ex. Pinus ponderosa Colorado n° 2863
ponderosae ex. Pinus ponderosa Colorado n° 2867
ponderosae ex. Pinus ponderosa Colorado n° 2872
ponderosae ex. Pinus ponderosa New Mexico n° 2924b
ponderosae ex. Pinus ponderosa Colorado n° 2871
ponderosae ex. Pinus ponderosa Colorado n° 2887
terminalis ex. Pinus edulis Colorado n° 2892
terminalis ex. Pinus edulis Colorado n° 2899bis
terminalis ex. Pinus monophylla Californie n° 2955
terminalis ex. Pinus monophylla Californie n° 2964
terminalis ex. Pinus monophylla Californie n° 2966
terminalis ex. Pinus edulis Colorado n° 2858
terminalis ex. Pinus edulis New Mexico n° 2922
pinivora ex. Pinus contorta Washington n° 3018
contortae ex. Pinus contorta Washington n° 3034
murrayanae ex. Pinus contorta Orégon n° 3071
murrayanae ex. Pinus contorta Orégon n° 3063
murrayanae ex. Pinus contorta Orégon n° 3067
medispinosa ex. Pinus contorta Colorado n° 2874
medispinosa ex. Pinus contorta Colorado n° 2875
medispinosa ex. Pinus contorta Colorado n° 2873
murrayanae ex. Pinus contorta Orégon n° 3051
contortae ex. Pinus contorta Orégon n° 3010
contortae ex. Pinus contorta Orégon n° 3050
murrayanae_ medispinosa ex. Pinus contorta Orégon n° 3065
murrayanae ex. Pinus contorta Orégon n° 3066
brevispinosa ex. Pinus radiata Californie n° 2988
brevispinosa ex. Pinus contorta Washington n° 3016
brevispinosa ex. Pinus contorta Washington n° 3040
brevispinosa ex. Pinus contorta Orégon n° 3060
pini ex. Pinus sylvestris Provence-Alpes-Côte d'Azur n° 2710
pini ex. Pinus sylvestris Provence-Alpes-Côte d'Azur n° 2729
pini ex. Pinus syvestris Provence-Alpes-Côte d'Azur n° 2934
pini ex. Pinus sylvestris Provence-Alpes-Côte d'Azur n° 2935
pini ex. Pinus sylvestris Provence-Alpes-Côte d'Azur n° 2932
pini ex. Pinus nigra Limousin n° 1261a
pini ex. Pinus sylvestris Provence-Alpes-Côte d'Azur n° 2682
maghrebica ex. Pinus halepensis Languedoc-Roussillon n° 1070
maghrebica ex. Pinus halepensis Languedoc-Roussillon n° 819
maghrebica ex. Pinus halepensis Khenchela n° 2382
palaestinensis ex. Pinus halepensis Languedoc-Roussillon n° 1046
palaestinensis ex. Pinus halepensis Péloponèse n° 1542
near_guadarramae ex. Pinus nigra Almaty n° 2829
nuda ex. Pinus pinaster Aquitaine n° 2837
schwartzi ex. Pinus ponderosa Colorado n° 2898
schwartzi ex. Pinus ponderosa New Mexico n° 2924c
schwartzi ex. Pinus ponderosa Colorado n° 2901b
schwartzi ex. Pinus ponderosa Colorado n° 2877
schwartzi ex. Pinus ponderosa Californie n° 2971
edulis ex. Pinus edulis Colorado n° 2891
edulis ex. Pinus edulis Colorado n° 2894
arizonica ex. Pinus ponderosa Californie n° 2995
arizonica ex. Pinus ponderosa Orégon n° 3044
pseudotaxifoliae ex. Pseudostuga sp New Mexico n° 2917
pseudotaxifoliae ex. Pseudostuga sp New Mexico n° 2919
pseudotaxifoliae ex. Abies sp New Mexico n° 2921
pseudotaxifoliae ex. Pseudotsuga ou Abies Colorado n° 2882
pseudotaxifoliae ex. Pseudotsuga sp Colorado n° 2888
pseudotaxifoliae ex. Pseudotsuga sp Orégon n° 3047
pseudotaxifoliae ex. Pseudotsuga sp Orégon n° 3056
pseudotaxifoliae ex. Pseudotsuga menziesii Orégon n° 3001
pseudotaxifoliae ex. Pseudotsuga sp Californie n° 2981
pseudotaxifoliae ex. Pseudotsuga sp Californie n° 2991
pseudotaxifoliae ex. Pseudotsuga sp Californie n° 2994
pseudotaxifoliae ex. Pseudotsuga sp Californie n° 3076
pseudotaxifoliae ex. Pseudotsuga menziesii Orégon n° 2999
pseudotaxifoliae ex. Pseudotsuga sp Washington n° 3029
pseudotaxifoliae ex. Pseudotsuga sp Washington n° 3041
splendens_sensu_Palmer ex. Pseudotsuga sp Californie n° 2987
splendens_sensu_Palmer ex. Pseudotsuga sp Orégon n° 3004
splendens_sensu_Palmer ex. Pseudotsuga macrocarpa Washington n° 3025
splendens_sensu_Palmer ex. Pseudotsuga menziesii Californie n° 2996
splendens_sensu_Palmer ex. Pseudotsuga sp Californie n° 3077
splendens_sensu_Palmer ex. Pseudotsuga sp Californie n° 3093
pseudotsugae_sensu_Palmer ex. Pseudotsuga ou Abies Colorado n° 2881
pseudotsugae_sensu_Palmer ex. Pseudotsuga sp Colorado n° 2889
pseudotsugae_sensu_Palmer ex. Pseudotsuga sp Colorado n° 2890
pseudotsugae_sensu_Palmer ex. Pseudotsuga sp New Mexico n° 2915bis
pseudotsugae_sensu_Palmer ex. Pseudotsuga sp New Mexico n° 2918
laricis ex. Larix decidua Valais n° 3094
laricis ex. Larix decidua Languedoc-Roussillon n° 445b
laricis ex. Larix decidua Provence-Alpes-Côte d'Azur n° 2945
laricis ex. Larix decidua Provence-Alpes-Côte d'Azur n° 2708
laricifoliae ex. Larix occidentalis Orégon n° 3058
laricifoliae ex. Larix occidentalis Orégon n° 3068
kochiana ex. Larix decidua Provence-Alpes-Côte d'Azur n° 2762
kochiana ex. Larix decidua Provence-Alpes-Côte d'Azur n° 2940
cuneomaculata ex. Larix decidua Rhône-Alpes n° 2612
cuneomaculata ex. Larix decidua Provence-Alpes-Côte d'Azur n° 2700
cuneomaculata ex. Larix decidua Provence-Alpes-Côte d'Azur n° 2692
cuneomaculata ex. Larix decidua Provence-Alpes-Côte d'Azur n° 2628
cuneomaculata ex. Larix decidua Provence-Alpes-Côte d'Azur n° 2693
cuneomaculata ex. Larix decidua Languedoc-Roussillon n° 445a
cuneomaculata ex. Larix decidua Rhône-Alpes n° 2601
cedri ex. Cedrus sp Sicile n° 1973
cedri ex. Cedrus sp Sicile n° 1974
cedri ex. Cedrus sp Washington n° 3033
cedri ex. Cedrus sp Sicile n° 1971
cedri ex. cedrus sp Languedoc-Roussillon n° 2802
cedri ex. cedrus sp Languedoc-Roussillon n° 2834
puerca ex. Pinus edulis Colorado n° 2895
puerca ex. Pinus monophylla Californie n° 2968
close_C_ piceae ex. Picea sp Colorado n° 2879
close_C_ piceae ex. Picea sp Colorado n° 2880
close_C_ piceae ex. Picea sp Californie n° 2972
close_C_ piceae ex. Picea sp New Mexico n° 2914
close_C_ piceae ex. Picea engelmannensis Colorado n° 2903a
close_coloradensis ex. Picea sp Orégon n° 3048
curtihirsuta ex. Abies concolor Californie n° 3072
curtihirsuta ex. Abies concolor Californie n° 3075
curtihirsuta ex. Abies sp Californie n° 3078
curtihirsuta ex. Abies grandis Orégon n° 3053
curtihirsuta ex. Abies sp Orégon n° 3070
wahtolca ex. Pinus edulis Colorado n° 2897
wahtolca ex. Pinus edulis Colorado n° 2900
wahtolca ex. Pinus edulis Colorado n° 2893
wahtolca ex. Pinus edulis New Mexico n° 2923
anelia ex. Pinus monophylla Californie n° 2956
anelia ex. Pinus monophylla Californie n° 2961
anelia ex. Pinus monophylla Californie n° 2967
moketa ex. Pinus lambertiana Californie n° 3085
moketa ex. Pinus lambertiana Californie n° 3080
moketa ex. Pinus lambertiana Californie n° 3086
moketa ex. Pinus lambertiana Californie n° 3087
moketa ex. Pinus lambertiana Californie n° 3084
apini ex. Pinus flexilis New Mexico n° 2920
apini ex. Pinus flexilis Colorado n° 2865
cembrae ex. Pinus cembra Provence-Alpes-Côte d'Azur n° 2706
cembrae ex. Pinus cembra Provence-Alpes-Côte d'Azur n° 2760
glabra ex. Pinus ponderosa New Mexico n° 2924a
glabra ex. Pinus ponderosa Californie n° 2969b
glabra ex. Pinus ponderosa Colorado n° 2870b
solitaria ex. Pinus ponderosa Colorado n° 2862b
solitaria ex. Pinus ponderosa Colorado n° 2870a
watsoni ex. Pinus radiata Californie n° 2975
watsoni ex. Pinus radiata Californie n° 2982
fresai ex. Cupressus sp Californie n° 2983
fresai ex. Cupressus sp Orégon n° 3003
fresai ex. Cupressus sp Californie n° 2993
fresai ex. Cupressus sp Washington n° 3038
fresai ex. Cypres ou Thuja sp Aquitaine n° 2841
fresai ex. Juniperus sp Haute-Normandie n° 1785
tujafilina ex. Thuja orientalis Languedoc-Roussillon n° 2462
tujafilina ex. Thuja sp Languedoc-Roussillon n° 2835
tujafilina ex. Chamaecyparis sp Sicile n° 1969
juniperi ex. Juniperus communis Provence-Alpes-Côte d'Azur n° 2709
juniperi ex. Juniperus sp. Aquitaine n° 640bis
juniperi ex. Juniperus communis Provence-Alpes-Côte d'Azur n° 2720
juniperi ex. Juniperus communis Provence-Alpes-Côte d'Azur n° 2721
juniperi ex. Juniperus communis Provence-Alpes-Côte d'Azur n° 2763
juniperi ex. Juniperus communis Provence-Alpes-Côte d'Azur n° 2952
petersoni ex. Juniperus communis Colorado n° 2905
confinis ex. Abies cephalonica Languedoc-Roussillon n° 2800
confinis ex. Abies cephalonica Languedoc-Roussillon n° 2801
confinis ex. Abies nordmanniana Bourgogne n° 2466
grande ex. Abies sp Colorado n° 2906
pectinatae ex. Abies sp Languedoc-Roussillon n° 2725
pectinatae ex. Abies alba Languedoc-Roussillon n° 2727
pectinatae ex. Abies alba Languedoc-Roussillon n° 446
occidentalis ex. Abies concolor Californie n° 3079
occidentalis ex. Abies concolor Californie n° 3090
occidentalis ex. Abies concolor Californie n° 3074
occidentalis ex. Abies concolor Californie n° 3081
occidentalis ex. Abies sp Washington n° 3024
occidentalis ex. Abies sp Orégon n° 3049
occidentalis ex. Abies concolor Colorado n° 2884
occidentalis ex. Pseudotsuga sp New Mexico n° 2916
englemanniensis_bonica ex. Picea sp Colorado n° 2904
englemanniensis_bonica ex. Picea sp Colorado n° 2908
lachnus ex. N N N n° 1101
lachnus ex. N N N n° 2664
trama ex. N N N n° 1211
tuberolachnus ex. N N N n° 2318
0.2
